# Awareness and Needs of Smoking Cessation Services for Female Emotional Laborers, Parcel Delivery Workers, Transportation Workers, and Construction Workers in South Korea

**DOI:** 10.3390/ijerph192215220

**Published:** 2022-11-18

**Authors:** Dahyeon Lee, Kang-Sook Lee, Haena Kim, Yeonwoo Lee, Mi-Ji Lee, Hyunkyung Lee, Jun-Pyo Myong, Hyekyeong Kim, Jakyoung Lee

**Affiliations:** 1 Department of Health Promotion, Graduate School of Public Health and Healthcare Management, The Catholic University of Korea, 222 Banpo-daero, Seocho-gu, Seoul 06591, Republic of Korea; 2 Department of Preventive Medicine, College of Medicine, The Catholic University of Korea, 222 Banpo-daero, Seocho-gu, Seoul 06591, Republic of Korea; 3 Seoul Tobacco Control Center, 222 Banpo-daero, Seocho-gu, Seoul 06591, Republic of Korea; 4 Korean Association on Smoking or Health, Seoul 07238, Republic of Korea; 5 Department of Occupational and Environmental Medicine, Seoul St. Mary’s Hospital, College of Medicine, The Catholic University of Korea, Seoul 06591, Republic of Korea; 6 Department of Health Convergence, Ewha Women’s University, Seoul 03760, Republic of Korea; 7 Graduate School of Public Health, Yonsei University, Seoul 03722, Republic of Korea

**Keywords:** smoking cessation services, female emotional laborers, parcel delivery workers, transportation workers, construction workers

## Abstract

Although South Korea has implemented various smoking cessation services, women who are emotional laborers, as well as parcel delivery, transportation, and construction workers, have poor access to these services. This study evaluated the smoking-related characteristics of workers in these four occupations as well as the awareness of and need for smoking cessation services. In total 808 workers in these four occupations aged 19 years and above were recruited nationwide and had their data analyzed. The participants’ age, marital status, number of work hours per week, job-related stress, age when they started smoking, average number of cigarettes a day, types of tobacco products, close relationships to others who smoke, number of attempts to quit smoking, plans to quit smoking, awareness of cessation services, prior utilization of cessation services, and need for cessation services were surveyed. Compared with parcel delivery workers, female emotional laborers and transportation and construction workers had more attempts to quit smoking, plans to quit smoking, and prior utilization of smoking cessation services, moreover, construction workers had a significantly lower awareness of smoking cessation services. Parcel delivery workers need smoking cessation programs, mobile applications to help them quit smoking, and improvements in their work environments. Cessation services and education should be promoted at workplaces and among managers.

## 1. Introduction

The Framework Convention on Tobacco Control (FCTC, 2005) adopted by the World Health Organization (WHO, 2005) defines countries’ duties to ensure that all people enjoy the highest attainable level of physical and mental health by enforcing tobacco regulations. It also recommends that countries consider key differences among various populations in terms of sex, socioeconomic status, and disability to reduce tobacco demands and facilitate smoking cessation [1]. In South Korea, the male smoking rate dropped from 51.7% in 2005 to 36.7% in 2018, while that of females increased from 5.7% in 2005 to 7.5% in 2018. Furthermore, compared with 2019, the coronavirus disease 2019 (COVID-19) pandemic led to a 5.4% increase in cigarette sales in 2020, with e-cigarette sales and utilization also rising [2].

Research also shows that female emotional laborers suffer from high levels of job-related stress, mostly because they are forced to engage in surface acting, that is, expressing emotions and attitudes that are expected of them irrespective of their actual emotions and attitudes. Their stress levels are generally high while surface acting [3,4]. In South Korea, the number of emotional laborers rose from 7.40 million in 2011 to 13.30 million in 2017 [5]. More than 40% of the women working in the emotional labor industry smoke cigarettes [6,7].

The COVID-19 pandemic also led people to use more delivery and parcel delivery services as a result of increased online shopping, late-night deliveries, and one-person households; this reality led to the expansion of the parcel delivery and other delivery industries. An estimate of the number of delivery workers is 390,000 as of 2020, the highest since 2013 [8,9]. Additionally, since the introduction of the delivery industry around 20 years ago, we have seen a 30-fold growth, with a great portion of this growth being due to and after the COVID-19 pandemic. A 2020 survey on health in work environments showed a smoking rate of 66.5% among parcel delivery drivers [8,9]. Nonetheless, along with the increased utilization of parcel delivery services, delivery-related accidents are also surging, albeit most parcel delivery drivers are special-contract laborers without proper legal protection. In the case of special-contract laborers, they do not have legal protection regarding working hours, holidays, and sick leave. In South Korea, parcel delivery workers tend to work for six consecutive days a week and for 12 h or more per week day; hence, they tend to work at least 70 h a week, which is far beyond the standard for chronic overwork [10]. In Canada, parcel delivery services have grown in size since the outbreak of COVID-19, but these workers are not easily accessible through research, are unstable, and are at an elevated risk of COVID-19 exposure [11].

Transportation workers are at risk for lung cancer and are a difficult group to access to promote smoking cessation [12]. Additionally, varenicline, a smoking cessation aid, should be prescribed with caution for transportation workers because it can induce drowsiness and fatigue [13]. Smoking cessation services, as opposed to drugs, are more suitable for this group of workers [13]. In South Korea, while the percentage of older adult transportation workers is growing rapidly (in 2015, workers aged ≥ 60 years accounted for 49.2% of all taxi drivers), their work schedules are not ideal for their age group, which has led to an increased rate of traffic accidents [14]. In South Korea, since most transportation workers show significant blood pressure elevation and their experience of acute cardiovascular diseases can lead to mass fatalities, special precautions are required during their work hours [15]. For transport workers with high blood pressure or metabolic syndrome, health check-ups should be conducted regularly and rest periods should be taken. In 2019, approximately 1.154 million workers were known to be in the transportation industry, with about 431,704 working in ground-passenger transport services (taxi, bus), and a 2018 survey revealed that 45.3% out of the 321 taxi drivers studied were current smokers in South Korea [16,17].

As for construction workers, they are occupationally exposed to poor respiratory conditions [18], and they tend to work in environments that are associated with elevated risks for lung cancer. Thus, there is a pressing need for measures to reduce carcinogenic exposure and implement smoking cessation programs in construction worksites [19]. Most construction workers generally exhibit a high level of job-related stress and experience time pressure during work, which hampers a proper utilization of their due break times [20]. In a 2020 survey of occupations with high rates of death from overwork (cerebrovascular and cardiovascular diseases), the number of construction workers increased by 59.4% compared to 2019 [21]. In July 2020, approximately 76,000 construction companies were operative in South Korea, and despite a small decline in the number of construction workers due to the COVID-19 pandemic, they still account for about 7.5% of all workers in the country, and nearly 60% of them have been identified as current smokers [9,22]. In general, the rate of work productivity lost due to the adverse health effects of smoking is more than 64% [23].

All of these aforementioned jobs—namely emotional labor, parcel delivery, transportation, and construction—are highly unstable. Since higher job stability is likely to be linked to better perceived health and welfare, these workers are considered to be in a vulnerable situation regarding their health [22]. They are also at a higher risk for developing chronic obstructive pulmonary disease [24]. Additionally, smoking rates increase as the business scale decreases and are higher among service workers and production-line workers than among white-collar workers [25]. Smoking rates are also higher among day laborers than among regular employees, and most construction workers are day laborers [25].

The South Korean government established smoking cessation clinics in public health centers in 2004, a quitline in 2006, in-person smoking cessation services, four-night/five-day residential programs, inpatient programs, and 17 smoking cessation centers nationwide in 2015. The four-night/five-day residential program entails a camp that provides psychological counseling and drug treatment [26].

However, occupations with high smoking rates, namely emotional labor, parcel delivery, transportation, and construction, are related to the low accessibility of these smoking cessation services. While some studies have examined smoking cessation among white-collar workers, few scholars have exclusively focused on occupations with a high smoking rate, as well as difficulties in accessing smoking cessation services and in quitting smoking. This study aimed to analyze the smoking-related characteristics of occupations with health vulnerabilities, such as women working as emotional laborers, parcel delivery workers, transportation workers, and construction workers, while identifying their awareness of and need for smoking cessation services.

## 2. Methods

### 2.1. Data Collection, Participants, and Setting

This study was conducted with 819 female emotional laborers, parcel delivery workers, transportation workers, and construction workers aged 19 years and above. After excluding 11 individuals with missing data, the final sample comprised 808 participants, including 201 female emotional laborers, 324 male parcel delivery workers, 106 male transportation workers, and 188 male construction workers. All emotional labor workers in our sample were women; all parcel delivery, transportation, and construction workers in this sample were men.

The questionnaire was administered to workers with prior utilization of smoking cessation services, relevant community users, members of Kakaotalk open chat groups for relevant workers, corporate-owned taxi drivers, and construction workers regarding safety education. The questionnaire included a section in which participants chose whether they consented to participate in the study, and all participants provided consent. The survey method was conducted in two ways: paper questionnaire and Google online form. Data were collected from 17 August 2021 to 15 October 2021. This study was approved by the Institutional Review Board at the Catholic University of Korea (MC21QASI0091).

### 2.2. Survey

#### 2.2.1. General Characteristics

The questionnaire contained questions on general characteristics, including age, marital status, number of work hours per week, and job-related stress. Age was divided into groups in 10-year units. Marital status was divided into being married, single, or separated. Number of work hours per week were defined as the average work hours per week. Job-related stress was assessed using eight questions rated on a Likert scale, and the scores were summed. Each item is composed of a 5-point Likert scale of ‘not at all’, ‘disagree’, ‘moderate’, ‘agree’, and ‘strongly agree’. The total possible score was 40, with higher scores indicating greater job-related stress. Cronbach’s α was measured to be 0.76.

#### 2.2.2. Smoking-Related Characteristics

Age when first starting smoking, average number of cigarettes a day, and thoughts about smoking were surveyed. Thoughts about smoking were assessed using six questions that followed a Likert scale, and the scores were summed for a total of 30. Each question was answered on a 5-point Likert scale ranging from ‘not at all’ to ‘strongly agree’. The higher the score, the more positive one’s thoughts about smoking. 

The specific tobacco products used, close relationships to others who smoke, and method of attempts to quit smoking were surveyed using multiple-choice questions. For the specific tobacco product used, e-cigarettes included nicotine-containing products and nicotine-free products; heated tobacco products included closed system vapes; and others included roll-your-own cigarettes, cigars and cigarillos, hookah, and snuff. Regarding method of attempts to quit smoking, participants’ prior attempts and future plans to quit smoking were surveyed.

#### 2.2.3. Awareness of Smoking Cessation Services 

Awareness of smoking cessation services included services that the participant had heard of or knew about, the channels through which they got to know about smoking cessation services, prior utilization of smoking cessation services, and individualized educational content in smoking cessation services. The participants were asked to choose all options applicable.

### 2.3. Statistical Analysis 

The data were analyzed using SPSS software version 28.0. Participants’ general characteristics, prior attempts to quit smoking, future plans to quit smoking, work environment features that were considered during individual counseling, and need for smoking cessation services were analyzed using crosstab analyses and chi-square tests. Number of work hours per week, job-related stress, and smoking-related characteristics were analyzed with one-way ANOVA and Scheffe’s test. Data on the specific tobacco product used, close relationships to others who smoke, method of attempts to quit smoking, and awareness and utilization of smoking cessation services were collected using multiple-choice questions and analyzed with crosstab analyses. 

Furthermore, prior attempts to quit smoking, future plans to quit smoking, awareness of smoking cessation services, and utilization of smoking cessation services were analyzed through binomial logistic regression, after adjusting for age and marital status with reference to the parcel delivery group, which had the lowest rates for these items and was considered to be the most vulnerable group of workers during the COVID-19 pandemic [8,9,11]. Prior attempts to quit smoking analyzed the binary logistic by dividing it into yes-or-no responses. Methods of attempting to quit smoking were analyzed by dichotomous logistic analysis, dividing them into cases in which the national smoking cessation service was used among the six options and the cases in which it was not. Regarding future plans to quit smoking in the binomial logistic regression, people with plans to do so within six months were classified as the Yes group; those with plans to quit smoking someday (not within six months) and without any plans to do so as of now were classified as the No group.

## 3. Results

### 3.1. Participants’ Characteristics

The predominant age group was that of 20–29-year-olds (*n* = 245, 30.3%). Regarding marital status, 380 (47%) of the participants were married, while 387 (47.9%) were single. The mean number of work hours per week was 44.81 h, and parcel delivery workers had the highest number of work hours per week (50.59 h), followed by transportation workers (46.43 h). The mean job-related stress score was 23.75 (out of 40), and parcel delivery workers had the highest level of job-related stress (24.92) (Table 1).

### 3.2. Smoking-Related Characteristics 

The mean age of starting smoking was 19.74 years. The group with the earliest age of starting smoking was that of the parcel delivery workers (19.16 years). The average number of cigarettes per day was 13.74. The heaviest-smoking group consisted of parcel delivery workers (16.33 cigarettes). The most common tobacco product used was cigarettes *(n* = 700, 86.6%), followed by liquid-based e-cigarettes (*n* = 139, 17.2%). Friends (*n* = 541, 67%), followed by coworkers (*n* = 483, 59.8%) were the groups people most commonly identified as being smokers. 

A total of 602 participants (74.5%) had attempted to quit smoking, with the highest number of attempts in the female emotional laborer group (*n* = 182, 90.5%) and the lowest number of attempts in the parcel delivery group (*n* = 122, 37.7%). The most common method of attempting to quit smoking was one’s own will (*n* = 512, 63.4%), followed by national smoking cessation services (*n* = 219, 27.1%). Moreover, 194 (24%) participants stated that they have plans to quit smoking within a month. This rate was the highest in the female emotional laborer group (*n* = 82, 40.8%) and lowest in the parcel delivery worker group (*n* = 35, 10.8%). A total of 143 (17.7%) participants stated that they have no plans to quit smoking, and this rate was the highest in the parcel delivery worker group (*n* = 66, 20.4%; Table 2).

### 3.3. Awareness of Smoking Cessation Services 

The highest percentage of participants (*n* = 676, 83.7%) had heard of or knew about smoking cessation clinics at public health centers, and the lowest percentage of participants (*n* = 87, 10.8%) knew about the four-night/five-day residential program. The most common channel to learn about smoking cessation services was through TV and radio advertisements (*n* = 342, 34.5%), followed by the Internet (*n* = 305, 30.8%). The smoking cessation service with the highest rate of utilization was smoking cessation clinics at public health centers (*n* = 267, 33%), followed by in-person smoking cessation services (*n* = 142, 17.6%). Regarding the individualized educational content in smoking cessation services, the most common response was receiving a worker-tailored gift after successfully quitting (*n* = 178, 22%; Table 3).

### 3.4. Need for Smoking Cessation Services 

In regards to the work environment features that should be considered during individual counseling, the most common response was having a poor rest area (*n* = 361, 44.7%), followed by irregular break times (*n* = 350, 43.3%). Regarding the important things to consider when providing smoking cessation services, the most common response was counseling for stress management (*n* = 200, 24.8%), followed by providing individualized products to motivate behavior and gifts for successfully quitting (*n* = 187, 23.1%). Regarding the topics that should be discussed in more detail during counseling, the most common response was stress control (*n* = 339, 42%), followed by withdrawal symptoms (*n* = 206, 25.5%). The preferred method of smoking cessation services was mobile applications (contactless; *n* = 430, 53.2%), followed by in-person smoking cessation services that are delivered at the participant’s location (face-to-face; *n* = 139, 17.2%; Table 4).

### 3.5. Comparison with Parcel Delivery Workers—The Most Vulnerable Group of Workers during the COVID-19 Pandemic

The odds ratio (OR) of the number of attempts to quit smoking was significantly higher among female emotional laborers (OR = 11.18, CI = 6.32–19.79), transportation workers (OR = 2.52, CI = 1.42–4.47), and construction workers (OR = 1.78, CI = 1.17–2.73) compared to parcel delivery workers. The OR of having future plans to quit smoking was significantly higher among female emotional laborers (OR = 5.43, CI = 3.57–8.26), transportation workers (OR = 3.42, CI = 2.14–5.46), and construction workers (OR = 2.27, CI = 1.52–3.40) compared to parcel delivery workers. The OR of the awareness of smoking cessation services was significantly lower among construction workers (OR = 0.40, CI = 0.18–0.91) compared to parcel delivery workers. The OR of having utilized smoking cessation services was significantly higher among female emotional laborers (OR = 8.81, CI = 5.60–13.86) and transportation workers (OR = 3.92, CI = 2.40–6.40) compared to parcel delivery workers (Table 5).

## 4. Discussion

In this study on occupations with high rates of smoking, the predominant age group was that of 20–29-year-olds. One’s early 20s is a period of young adulthood, and smoking during this time can have a lasting impact on one’s life. During adolescence and one’s early 20s, the brain is undergoing quick development and is more vulnerable to nicotine addiction [27]. A growing number of young adults begin smoking at a younger age, and the adverse outcomes of addiction manifest in older adulthood [28]. Smoking cessation services and campaigns to quit smoking could target young adults in their early 20s, with smoking cessation education beginning in adolescence.

Regarding the number of work hours per week, the parcel delivery drivers worked the longest hours, and they also had the greatest level of job-related stress, highlighting the need to improve their work environment. Since a high level of job-related stress influences one’s smoking and nicotine dependence, work environments should be ameliorated to reduce workers’ levels of job-related stress [29,30]. Further, this is consistent with previous findings stating that the smoking rate increases with increasing work hours [25]. Another study reported that smokers displayed a high level of job-related stress, rarely engaged in health-promoting behaviors, and smoked to reduce their job-related stress [7]. Stress management should be a primary focus of smoking cessation services. A previous study found that smoking cessation success rates increase with an increase in the number of counseling sessions, as counseling is focused on stress management [31]. In light of these results, the number of counseling sessions could be increased in smoking cessation programs to focus on stress management.

Cigarettes were the most common type of tobacco product used, and e-cigarettes were the second most common. E-cigarettes have consistently become more popular in recent years. Since the use of and dependence on e-cigarettes differ from those for conventional cigarettes, a different approach must be taken in cessation efforts [32]. E-cigarettes can have an array of effects on nicotine delivery due to the differences in the liquids, components, hardware, and user behavior. The scope of regulations for e-cigarettes should be broadened to include regulations on nicotine dosage [33]. 

Regarding their relationships to other smokers, female emotional laborers and construction workers stated that their friends were often smokers, while parcel delivery workers and transportation workers stated that their coworkers were smokers. The surrounding social environment is hence likely to influence smoking initiation and cessation in adolescents and young adults. It may be more effective for smoking cessation education to target smoking among peers [34]. That is, providing smoking cessation programs within workplaces may help lower workers’ smoking rates. 

The smoking cessation service with which participants were the most familiar was smoking cessation clinics at public health centers, followed by quitline [35]. The most common channels through which workers encountered smoking cessation services included TV and radio advertisements. Previous studies reported that providing information about health and educating workers regarding promoting health within workplaces are effective methods of health education [35]. Running advertisements for smoking cessation services through the media present in workers’ lounges, namely on TVs and radios, would be effective.

The work environment features that should be the most considered during individual counseling based on our sample were poor-quality rest environments, followed by irregular break times. A prior study reported that providing safety and smoking cessation education at work sites through a lunch truck was effective in workplaces with irregular break times and poor-quality rest environments [35]. This shows that smoking cessation and stress management in-person services could be developed for parcel delivery, transportation, and construction workers. 

The topic that should be discussed in the greatest depth during counseling according to participants was stress management, calling for a deeper focus on this topic in counseling for smoking cessation than what is currently discussed in related services. The type of smoking cessation service that our participants preferred was mobile applications (contactless). Previous studies reported that using a self-monitored app to quit smoking led to successful quitting as determined through the carbon monoxide (CO) level [36], and sending motivating text messages to assist with quitting smoking helped individuals to successfully quit [37]. Indeed, mobile applications for quitting smoking are anticipated to be useful during the COVID-19 pandemic due to their contactless nature.

Parcel delivery workers showed lower rates of attempts and plans to quit smoking compared to the other occupations, and they also displayed a lower awareness and prior utilization of smoking cessation services. One way to improve these rates and promote smoking cessation services may be to educate their respective managers. It has been proven that educating managers on smoking cessation service promotion or cessation interventions is a sustainable strategy for implementing related services [38]. Since the age of starting smoking, motivation to quit smoking, information about smoking cessation services, work environments, and job-related stress differ across occupations, workplace smoking cessation services or occupation-specific services could be developed [39].

While white-collar workers are likely to be influenced by tobacco regulations and have relatively greater access to information about smoking cessation services than blue-collar workers, the latter face difficulties in curtailing their smoking and do not show marked changes in their smoking behaviors [40]. Fostering anti-smoking workplace environments for blue-collar occupations is an urgent agenda. A previous study reported that involving managerial positions and relevant workers was significantly helpful for developing and promoting smoking cessation services within workplaces [41]. New occupation-specific smoking cessation services tailored to South Korean society could be developed with reference to the findings of applicable studies. Occupations vulnerable to smoking should be given an increased access to smoking cessation services in order to prevent any blind spots in their provision.

### Limitations

This study had a few limitations. First, as the participants were recruited from smokers who had utilized face-to-face smoking cessation services, their acquaintances, and members of some online communities, the findings of this study cannot be generalized to the general public. Second, we used a self-report questionnaire about the participants’ past experiences, entailing the possibility of recall bias. Third, the occupations examined in this study were confined to female emotional labor, parcel delivery, transportation, and construction. Subsequent studies should examine more vulnerable occupations in terms of health. Finally, another limitation is that the number of female emotional workers was 201, and there was no other female occupation group. Emotional labor is paid labor that manages and controls one’s emotions. They have a strong labor force and a high smoking rate. There are smoking cessation support services for this occupational group, but the usage rate is low, so the smoking characteristics of this occupational group and the demand for smoking cessation support services are factors that should be investigated. This study was conducted only for the occupational groups with high rates of smoking and high levels of difficulty in quitting smoking.

## 5. Conclusions

Parcel delivery workers rarely attempted to quit smoking, rarely had plans to quit smoking, and had little knowledge about or experience with smoking cessation services compared with female emotional laborers, transportation workers, and construction workers. Smoking cessation services tailored for parcel delivery workers should be designed and their work environments should be improved, including regular break times and shorter work hours. Smoking cessation services could focus more on stress management. Furthermore, smoking cessation services could be promoted within workplaces, and relevant education as well as services could be promoted among managerial positions.

## Figures and Tables

**Table 1:** Participants’ general characteristics.

		Female Emotional Laborer	Parcel Delivery Worker	Transportation Worker	Construction Worker	Total	Chi-Square	*p*-Value
		N = 201 *n* (%)	N = 324 *n* (%)	N = 106 *n* (%)	N = 177 *n* (%)	N = 808 *n* (%)		
Age	20–29	139 (69.2)	56 (17.3)	10 (9.4)	40 (22.6)	245 (30.3)	238.8	0.000
	30–39	32 (15.9)	121 (37.3)	31 (29.2)	49 (27.8)	233 (28.8)		
	40–49	13 (6.5)	97 (29.9)	28 (26.4)	46 (26.1)	184 (22.8)		
	50–59	15 (7.5)	49 (15.1)	28 (26.4)	28 (15.8)	120 (14.9)		
	60 over	2 (1)	1 (0.3)	9 (8.5)	14 (7.9)	26 (3.2)		
Marital status	Married	31 (15.4)	195 (60.2)	70 (66)	84 (47.5)	380 (47)	124.8	0.000
	Single	158 (78.6)	116 (35.8)	31 (29.2)	82 (46.3)	387 (47.9)		
	Separated	12 (6)	13 (4)	5 (4.6)	11 (6.3)	41 (5.1)		
Number of work hours per week (mean ± SD)		34.45 ± 16.43 ^a^*	50.59 ± 17.23 ^c^	46.43 ± 14.83 ^bc^	43.48 ± 15.69 ^b^	44.81 ± 17.52		0.000
Job-related stress (mean ± SD)		23.21 ± 5.03 ^ab^	24.92 ± 4.57 ^c^	23.75 ± 4.50 ^bc^	22.28 ± 5.10 ^a^	23.76 ± 4.90		0.000

* Same letters indicate an insignificant difference.

**Table 2:** Participants’ smoking-related characteristics.

		Female Emotional Laborer	Parcel Delivery Worker	Transportation Worker	Construction Worker	Total	Chi-Square	*p*-Value
		N = 201 *n* (%)	N = 324 *n* (%)	N = 106 *n* (%)	N = 177 *n* (%)	N = 808 *n* (%)		
Age when they first started smoking (mean ± SD)		20.26 ± 4.85	19.16 ± 2.98	20.24 ± 4.66	19.94 ± 5.21	19.74 ± 4.28		0.013
Cigarettes per day (mean ± SD)		7.78 ± 6.35 ^a^*	16.33 ± 8.41 ^c^	13.33 ± 7.68 ^b^	15.32 ± 8.23 ^bc^	13.74 ± 8.53		0.000
Thoughts about smoking (mean ± SD)		16.31 ± 3.64	16.35 ± 3.53	15.85 ± 4.19	15.75 ± 4.15	16.14 ± 3.79		0.277
Type of tobacco product (Multiple choice)	Cigarette (traditional)	166 (82.6)	293 (90.4)	87 (82.1)	154 (87)	700 (86.6)		
	E-cigarette	61 (30.4)	33 (10.1)	24 (22.6)	21 (11.9)	139 (17.2)		
	Heated tobacco product	34 (16.9)	41 (12.6)	13 (12.2)	20 (11.3)	108 (13.3)		
	Others	7 (3.5)	3 (0.9)	0 (0)	4 (2.3)	14 (1.7)		
Close relationships to others who smoke (Multiple choice)	Parents	57 (28.4)	84 (25.9)	21 (19.8)	35 (19.8)	197 (24.4)		
	Brother/sister	46 (22.9)	74 (22.8)	23 (21.7)	41 (23.2)	184 (22.8)		
	Spouse/lover	44 (21.9)	18 (5.6)	13 (12.3)	12 (6.8)	87 (10.8)		
	Children/grandchildren	4 (2)	4 (1.2)	6 (5.7)	9 (5.1)	23 (2.8)		
	Friends	148 (73.6)	224 (69.1)	61 (57.5)	108 (61)	541 (67)		
	Co-worker	72 (35.8)	239 (73.8)	70 (66)	102 (57.6)	483 (59.8)		
	Others	18 (9)	24 (7.4)	3 (2.8)	26 (14.7)	71 (8.8)		
Method of attempts to quit smoking (Multiple choice)	One’s own will	153 (76.1)	173 (53.4)	68 (64.2)	118 (66.7)	512 (63.4)		
	Nicotine substitutes	21 (10.4)	42 (13)	19 (17.9)	23 (13)	105 (13)		
	Internet, portal site	8 (4)	9 (2.8)	5 (4.7)	9 (5.1)	31 (3.8)		
	National smoking cessation services	107 (53.2)	49 (15.1)	38 (35.8)	25 (14.1)	219 (27.1)		
	Treatment in hospital (medication)	7 (3.5)	11 (3.4)	10 (9.4)	9 (5.1)	37 (4.6)		
	Others	19 (9.5)	124 (38.3)	18 (17)	48 (27.1)	209 (25.9)		
Prior attempts to quit smoking	Yes	182 (90.5)	202 (62.3)	88 (83)	130 (73.4)	602 (74.5)	56.7	0.000
	No	19 (9.5)	122 (37.7)	18 (17)	47 (26.6)	206 (25.5)		
Plans to quit smoking	Within one months	82 (40.8)	35 (10.8)	36 (34)	41 (23.3)	194 (24)	84.5	0.000
	Within six months	35 (17.4)	45 (13.9)	20 (18.9)	31 (17.5)	131 (16.2)		
	Someday	63 (31.3)	178 (54.9)	33 (31.1)	66 (37.3)	340 (42.1)		
	No plan	21 (10.4)	66 (20.4)	17 (16)	39 (22)	143 (17.7)		

* Same letters indicate an insignificant difference.

**Table 3:** Awareness of smoking cessation services.

		Female Emotional Laborer	Parcel Delivery Worker	Transportation Worker	Construction Worker	Total
		N = 201 *n* (%)	N = 324 *n* (%)	N = 106 *n* (%)	N = 177 *n* (%)	N = 808 *n* (%)
Services that the participant has heard of or knows about (Multiple choice)	Smoking cessation clinics at public health centers	159 (79.1)	275 (84.9)	91 (85.8)	151 (85.3)	676 (83.7)
	Quitline	101 (50.2)	152 (46.9)	39 (36.8)	74 (41.8)	366 (45.3)
	Four-night/five-day residential program	32 (15.9)	34 (10.5)	8 (7.5)	13 (7.3)	87 (10.8)
	In-person smoking cessation service	106 (52.7)	34 (10.5)	8 (7.5)	15 (8.5)	163 (20.2)
	Treatment in hospital	53 (26.4)	133 (41)	40 (37.7)	58 (32.8)	284 (35.1)
	Doesn’t know (never heard of)	6 (3)	11 (3.4)	8 (7.5)	15 (8.5)	40 (5)
The channel through which they got to know about smoking cessation services (Multiple choice)	TV and radio advertisements	36 (17.9)	141 (43.5)	46 (43.4)	83 (47.2)	342 (34.5)
	Placards, posters, brochures	52 (25.9)	69 (21.3)	22 (20.8)	44 (25)	211 (21.3)
	The Internet	50 (24.9)	138 (42.6))	28 (26.4)	56 (31.8)	305 (30.8)
	Public health center information	32 (15.9)	87 (26.9)	37 (34.9)	51 (29)	274 (27.6)
	Recommendations	32 (15.9)	83 (25.6)	22 (20.8)	46 (26.1)	229 (23.1)
	Through their company	101 (50.2)	16 (4.9)	21 (19.8)	16 (9.1)	169 (17.1)
	Medical advice	6 (3)	15 (4.6)	7 (6.6)	8 (4.5)	52 (5.2)
	Others	24 (11.9)	13 (4)	8 (7.5)	10 (5.6)	81 (8.2)
Prior utilization of smoking cessation services (Multiple choice)	Smoking cessation clinics at public health centers	50 (24.9)	101 (31.2)	55 (51.9)	61 (34.5)	267 (33)
	Quitline	27 (13.4)	19 (5.9)	13 (12.3)	19 (10.7)	78 (9.7)
	Four-night/five-day residential program	1 (0.5)	1 (0.3)	3 (2.8)	2 (1.1)	7 (0.9)
	In-person smoking cessation service	106 (52.7)	6 (1.9)	19 (17.9)	11 (6.2)	142 (17.6)
	None	41 (20.4)	201 (62)	29 (27.4)	94 (53.7)	366 (45.3)
Individualized educational content in smoking cessation services (Multiple choice)	Gift for quitting success	79 (39.3)	35 (10.8)	33 (31.1)	31 (17.5)	178 (22)
	Job-related stress counseling	15 (7.5)	20 (6.2)	14 (13.2)	20 (11.3)	69 (8.5)
	Counseling for alcohol or health care	17 (8.5)	19 (5.9)	28 (26.4)	22 (12.4)	86 (10.6)
	Flexible management for irregular work patterns	5 (2.5)	4 (1.2)	7 (6.6)	3 (1.7)	19 (2.4)
	Social skills for refusing to smoke	7 (3.5)	15 (4.6)	11 (10.4)	15 (8.5)	48 (5.9)
	Physical and psychological changes after quitting smoking	26 (12.9)	25 (7.7)	17 (16)	14 (7.9)	82 (10.1)
	No experience	63 (31.3)	68 (21)	16 (15.1)	30 (16.9)	177 (21.9)
	Others	29 (14.4)	169 (52.2)	20 (18.9)	72 (40.7)	290 (35.9)

**Table 4:** Need for smoking cessation services.

		Female Emotional Laborer	Parcel Delivery Worker	Transportation Worker	Construction Worker	Total	Chi-Square	*p*-Value
		N = 201 *n* (%)	N = 324 *n* (%)	N = 106 *n* (%)	N = 177 *n* (%)	N = 808 *n* (%)		
Work environment features that should be considered during individual counseling (multiple choice)	Irregular break times	73 (36.3)	168 (51.9)	45 (42.5)	64 (36.2)	350 (43.3)		
	High smoking rates	105 (52.2)	119 (36.7)	36 (34)	60 (33.9)	320 (39.6)		
	Poor resting area	66 (32.8)	166 (51.2)	45 (42.5)	84 (47.5)	361 (44.7)		
	Bonds between fellow smokers	89 (44.3)	111 (34.3)	48 (45.3)	57 (32.2)	305 (37.7)		
	Others	5 (2.5)	4 (1.2)	0 (0)	2 (1.1)	11 (1.4)		
Important things to consider when providing smoking cessation services	Gift for quitting success	44 (21.9)	62 (19.1)	24 (22.6)	57 (32.2)	187 (23.1)	103.1	0.000
	Stress management	50 (24.9)	91 (28.1)	21 (19.8)	38 (21.5)	200 (24.8)		
	Risk of smoking-related diseases	9 (4.5)	40 (12.3)	11 (10.4)	27 (15.3)	87 (10.8)		
	Positive pressure from peer	25 (12.5)	26 (8)	17 (16)	25 (14.1)	93 (11.6)		
	Weight management	16 (8)	12 (3.7)	1 (0.9)	0 (0)	29 (3.6)		
	Withdrawal symptoms control	20 (10)	67 (20.7)	21 (19.8)	20 (11.3)	128 (15.8)		
	Refusing skills to smoke	8 (4)	8 (2.5)	8 (7.5)	5 (2.8)	29 (3.6)		
	Guaranteed anonymity	21 (10.4)	10 (3.1)	0 (0)	2 (1.1)	33 (4.1)		
	Flexibility to program time	8 (4)	8 (2.5)	3 (2.8)	3 (1.7)	22 (2.8)		
Topics that should be discussed in more detail during counseling	Stress management	95 (47.3)	131 (40.4)	40 (37.7)	73 (41.2)	339 (42)	33.4	0.004
	Refusing to smoke	14 (7)	27 (8.3)	15 (14.2)	15 (8.5)	71 (8.8)		
	Alcohol drinking control	18 (9)	28 (8.6)	8 (7.6)	19 (10.7)	73 (9)		
	Body changes after quitting	44 (21.9)	41 (12.7)	9 (8.5)	25 (14.1)	119 (14.7)		
	Withdrawal symptoms	30 (14.9)	97 (29.9)	34 (32.1)	45 (25.4)	206 (25.5)		
The preferred type of smoking cessation service	Mobile application (contactless)	114 (56.7)	171 (52.8)	52 (49.1)	93 (52.5)	430 (53.2)	36.7	0.001
	Quitline (contactless)	37 (18.4)	33 (10.2)	12 (11.3)	22 (12.4)	104 (12.9)		
	In-person smoking cessation service (face-to-face)	35 (17.4)	61 (18.8)	24 (22.6)	19 (10.7)	139 (17.2)		
	Smoking cessation clinics	10 (5)	44 (13.6)	11 (10.4)	26 (14.7)	91 (11.3)		
	Short-term intensive program	4 (2)	12 (3.7)	6 (5.7)	11 (6.2)	33 (4.1)		
	Others	1 (0.5)	3 (0.9)	1 (0.9)	6 (3.4)	11 (1.4)		

**Table 5:** Comparisons with parcel delivery workers as the most vulnerable group.

	Attempts to Quit	*p*-Value	Plans to Quit	*p*-Value	Awareness of Smoking Cessation Services	*p*-Value	Utilization of Smoking Cessation Services	*p*-Value
	OR (95% CI) *		OR (95% CI) *		OR (95% CI) *		OR (95% CI) *	
Parcel delivery workers	1		1		1		1	
Female emotional laborers	11.18 (6.32–19.79)	0.000	5.43 (3.57–8.26)	0.000	1.54 (0.54–4.41)	0.419	8.81 (5.60–13.86)	0.000
Transportation workers	2.52 (1.42–4.47)	0.002	3.42 (2.14–5.46)	0.000	0.40 (0.15–1.04)	0.060	3.92 (2.40–6.40)	0.000
Construction workers	1.78 (1.17–2.73)	0.008	2.27 (1.52–3.40)	0.000	0.40 (0.18–0.91)	0.028	1.40 (0.95–2.05)	0.086

* age, marriage adjusted odds ratio.

## Data Availability

Data sharing is not applicable to this article. All data generated or analyzed during this study are included in this published article. https://eirb.cmcnu.or.kr/irb.do (accessed on 16 October 2021).
